# Supplementary material for: The alignment of companies' sustainability behavior and emissions with global climate targets
Source: Nat Commun. 2023 Dec 5;14:7831. doi: 10.1038/s41467-023-43116-2 (PMC10698019; doi:10.1038/s41467-023-43116-2)
Supplement: Supplementary file 1 — Supplementary Information [file 41467_2023_43116_MOESM1_ESM.pdf]

Supplementary Information: The alignment of companies' sustainability behavior and emissions with global climate targets

Simone Cenci[1✉], Matteo Burato[1], Marek Rei[1,2] and Maurizio Zollo[1✉]

[1]*Leonardo Centre on Business for Society, Imperial College Business School, London, SW7 2BX, UK;*

[2]*Department of Computing, Imperial College London, London, SW7 2BX, UK*

A Details of the machine learning system for initiative detection

The company reports are analysed using automated systems which process the text and extract information about the initiatives described in each report. The system works in three stages: preprocessing, initiative detection and initiative classification. Below we provide a description of the system. Further details can be found in Ref. (1). Figure S1 provides a schematic representation of the workflow of the data-generating process.

A.1 Preprocessing

The company reports are in pdf format and the first step is to convert them to plain text. This is done using the Unix *pdftotext* tool.[¶] The text is then broken into individual sentences using the spaCy[*] toolkit and saved as a json file for further processing. Various metadata about the pdf, such as the creation time and an *md5* fingerprint, are also retained.

A.2 Initiative detection

Next, the reports are processed in order to detect the sustainability initiatives they describe. We trained supervised machine learning models to classify individual sentences as either belonging to an initiative description or not. We use two neural transformer models for initiative detection, and then combine their output probabilities in order to create an ensemble prediction with increased reliability. The models are trained on manually annotated company reports, with 507 reports in the training set and 81 reports in the development set.

The first model is based on *BERT-base* (2) and takes three sentences as input when making a prediction: the target sentence, the preceding sentence and the following sentence. This contextual information allows the model to better understand the meaning of the target sentence and make more accurate decisions. The second model is based on *RoBERTa-base* (3) and takes two sentences as input: the target sentence and the preceding sentence. As the RoBERTa model is larger by itself, we found it was sufficient to only give it one sentence of context as input.

✉To whom correspondence may be addressed. Email: s.cenci@imperial.ac.uk, m.zollo@imperial.ac.uk

¶https://linuxappfinder.com/package/poppler-utils

*https://spacy.io/

Both models were trained for 5 epochs on the annotated training data, using batch size 32 and learning rate $1e-05$. Early stopping of the training process was performed based on the sentence-level $F_{0.5}$ metric on the development set. The models were implemented using PyTorch (4) and Hugging Face (5).

We apply length filters, classifying any sentences shorter than 10 tokens or longer than 100 tokens as non-initiative. Most of such sentences are either numerical values extracted from tables or difficult cases for sentence separation. We found that only a small fraction of real initiatives have sentences with such extreme lengths, therefore the filtering is able to decrease processing time for the models with minimal decreases in accuracy. All the other sentences are passed through both machine learning models, the two predictions are averaged and any sentence with a score higher than 0.66 is classified as belonging to an initiative. This threshold was chosen based on evaluation on the development set and manual evaluation of a random output sample. We have set a threshold (0.66) that resulted in a 95% precision in the identification of the initiatives. We have tuned the algorithm to maximise precision because we wanted to ensure that the final dataset included only actions effectively implemented by firms in our sample. Any consecutive sentences that have been classified as belonging to an initiative are then combined into the same multi-sentence initiative.

A.3 Initiative classification

The output of the previous step provides spans of sentences that refer to initiatives described in a particular report. As the next step, we classify the initiatives based on three attrubutes: the SDG corresponding to that initiative, the type of the initiative and the stakeholder of the initiative.

For detecting each of these attributes we trained separate multi-class class classifiers based on *RoBERTa-base*. These models were given three sentences of input: the target, the preceding and the following sentence. The training was performed in batches of 32 for 20 epochs using a learning rate $1e-05$. Early stopping was performed based on the multi-class accuracy metric on the development set. The models predict a probability distribution showing the likelihood of each sentence belonging to a particular class. For multi-sentence initiatives, these distributions were averaged in order to produce a single probability distribution for the whole initiative. The classes with the highest average scores were then chosen as the final predictions for the SDG, initiative type and stakeholder of each initiative.

A.4 Performance on a test set

There are two important statistics to assess the performance of the algorithm in the test set: (1) the capacity to identify an initiative (recall), and (2) the quality of the classification (precision). In the validation set, we found a recall of $\sim 40\%$, i.e. that is we identify $\sim 40\%$ of the initiatives actually reported in the reports. The precision is $\sim 96\%$, i.e. 96% of the time we identify an initiative we were correct to classify the sentence as such. To assess the recall on the test set we manually read 20 reports and we calculate the ratio of initiatives identified manually and by the algorithm. Importantly, in the performance assessment test, we have used the same definition provided to the annotators to classify an initiative:

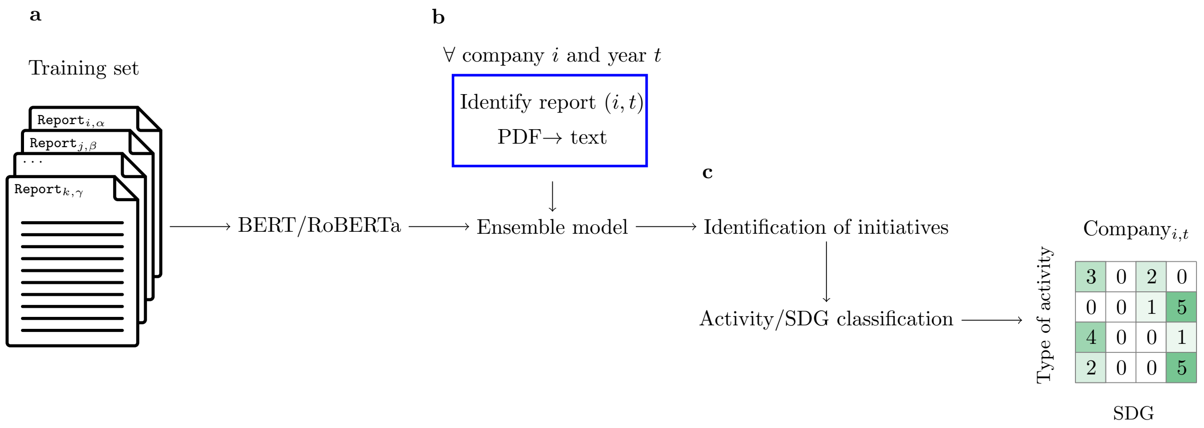

Fig. S1 Schematic representation of the data generating process. The data-generating process is divided into three steps. First, we used a manually annotated training set to build an ensemble model made of a BERT and RoBERTa based model (**a**). Second, we buy/crawl sustainability reports for every company i and year t in our sample and we transform the PDF into text (**b**). Finally, we apply the model to identify initiatives from the report and classified them based on the activity type and the SDG (**c**). The final dataset for company i in year y is a behavioural matrix where each entry counts the total number of initiatives in the particular Activity/SDG combination.

an initiative is an action that a company has done, or is actively pursuing. We have found that the algorithm has a recall of approximatively 24% in the test set (and precision of $\sim 94\%$). However, the recall is consistent across reports since the training set was chosen to reduce sector biases by sampling reports uniformly across sectors. Therefore, while the recall is low it is the same across companies and firm-by-firm comparisons are meaningful. Some company-specific bias is possible due to the format of the pdfs. To evaluate the capacity of the algorithm to classify initiatives into activities and SDGs we perform a manual check on a random sample of reports on the test set. Specifically, we randomly sample 300 initiatives and we assess the precision in the classification of the SDG, the activity and the joint activity/SDG precision. The results are shown in the table below. For illustrative purposes, in section C we report a sample of initiatives alongside their classification into activities and SDGs. Before showing the examples, in the next section, we present the definition of activities used in the analysis.

	Activity	SDG	Joint
Precision	87.2%	86.4%	78.6%

B Definition of sustainability activities

Here we provide the definition of the sustainability activities used in the main text.

- **Communications**: Activities that bring specific information or knowledge from the company to a certain interlocutor, to generate awareness, engage stakeholders, communicate policies, meetings and conferences, marketing campaigns and information about products, even through web communication.

- **Association**: Activities through which companies join, collaborate or promote cooperation with other companies, organisations, institutions or communities, including multilateral agreements and collaboration initiatives.
- **Donations & Funding**: Philanthropic activities through which companies donate money, goods or services as gifts. Includes supporting or sponsoring external sustainability-related organisations, initiatives or programs. In addition, it includes employee benefits, such as healthcare plans. (The donation comes from the corporate itself, not from its stakeholders.)
- **Volunteerism**: Activities that stimulate and promote volunteerism, fundraising and personal donations from individuals within or outside the company (i.e. employees, costumers, community volunteerism). Notably, employees donations of goods are encapsulated within Donation, unless the initiative's description specifies otherwise.
- **Adoption of Standards & Rules**: Activities involving the underwriting, adoption or compliance with externally sourced policies, guidelines, procedures, or standards.
- **Modification of Procedures**: Activities that modify the procedures adopted by the company in order to perform a specific activity (e.g. HR selection processes and supply chain activities).
- **Assessment and measurement**: Activities with which the company collect information from inside or outside. Including retrieval, research, survey, data collection, studies and measurement.
- **Organisational Structuring**: Activities that involve a structural change in the organisational structure of the company. Including the modification or establishment of new divisions, functions, roles (e.g. management positions), committees, teams or bodies.
- **Training**: Teaching activities aimed at improving knowledge, skills, and competencies.
- **Pricing**: Marketplace activities by which the company sets up or modifies pricing structures and tariffs.
- **Incentives**: Activities that typically involve the development of benefits, privileges, or rewards toward a particular stakeholder in order to gratify or stimulate an action. They might conversely take the form of active disincentives and punishments in order to discourage a detrimental action.
- **R&D Investments**: Activities that encompass an investment aimed at introducing a technological novelty in a product, service or process. They include structural investments in prototyping, trial and researching.
- **New product**: Launch of a new product or service (made available to the market). It includes new product's technical specification, the inclusion of new components or features into an existing product or service, as well as packaging.
- **Assets Modification**: Activities that build, expand or modify the physical assets owned and used by the Company to run their activities. This may include production assets, commercial assets and distribution assets (e.g. machines, devices, vehicles, buildings or facilities).

C Examples of sustainability initiatives

Here we provide a few examples of extracts from the texts of the sustainability initiatives.

Activity	SDG	Text
assessment & measurements	7	In order to better understand our energy consumption patterns and enhance the system efficiency of our buildings , we have commenced a three - year monitoring - based commissioning programme for the HVAC systems in [location] and [location] in [location] .
r&d investments	7	In this experiment , hydrogen is produced by the electrolysis of water with clean electricity generated from photovoltaic cells , which is then used to run three 5kW pure hydrogen fuel cell batteries. The aim of this experiment is to verify reliability and efficient operational control under variable power demands.Through this demonstration experiment , we aim to improve the pure hydrogen fuel cell functions , contributing to the creation of a society where people can live safely with clean energy.
association	11	Additionally, [Company] carries out various projects with third sector entities to provide training on efficiency and optimising the electricity bill , also reinforcing security measures and risk prevention for vulnerable families .
new products	12	[Company] is helping communities enhance air quality with [product] , a new class of low - emission yard locomotives. Branded [brand] for their operating efficiencies in reducing emissions and fuel use , the locomotives were funded in part by federal [policy] grants .
r&d investments	13	[Project] : Quantifying and Deploying Responsible Negative Emissions in Climate Resilient Pathways , a European Horizon 2020 Programme , to assess the realistic potential of Negative Emission Technologies and Practices (NETPs) and their contribution to climate neutrality , as a supplementary strategy to emissions mitigation.
asset modification	14	Hydroelectric power stations generate environment-friendly electricity , but can prevent migratory fish swimming upstream. When building new weirs such as that at [location] on the [location] in [location] or modernising such river power plant, [company] always installs fish ladders to ensure that the rivers remain passable for indigenous fish populations. Two more hydroelectric power stations were retrofitted with such ladders in 2010.
donation&funding	15	In 2020 trees were planted in the region of [location] .In a partnership established with [Organisation] five hectares of pine forest destroyed in the 2017 fires were rehabilitated , with the use of a donation amounting to 50 thousand obtained via the commitment of donating one euro per each client that subscribes to the e - statement instead of the paper one .This campaign enabled not only reduce the emissions by means of the reduction in the use of paper but also through the carbon storage achieved by the planted trees . [Company] , within the scope of its environmental policy and the Lisbon European Green Capital 2020 commitment compensated the emissions of GHE associated with an internal event , which accounted 67,0 tCO2e , through the certified forestation of an area in [location] .

Activity	SDG	Text
Asset modification	12	Since 2016 , we have replaced more than 14% of our fleet with more environmentally efficient vehicles , liquidating almost 1,000 vehicles and purchasing nearly 550 new vehicles .
Asset modification	7	This year , by using biogas as an emission - friendly renewable energy source , we have optimized the biogas plant to produce 1,5 megawatts of electricity .
Asset modification	7	In addition , at the commercial facility [location] , two wind turbines have been installed on the rooftop to foster the use of natural energy .
Asset modification	7	On the one hand , [Company] completed the upgrading of energy - saving heating pipes technology , LED lighting , solar power water heater and insulation materials to eliminate and replace equipment of high consumption and low energy efficiency by applying new energy - saving technologies and devices on production equipment and public facilities .
Association	7	In 2012 , we entered into a partnership with [location] - based [organisation], whose water electrolysis technology can convert surplus renewable energy into hydrogen gas .
Association	12	[Company] takes its responsibility to the environment very seriously .For this reason , it made the decision to participate in the “ Greenhouse Gas Reduction Programme ” .The aim of this initiative is to implement a 10% reduction in the greenhouse emissions which arise by the year 2012 .This is also a means to help achieve the targets set by the Kyoto Protocol in regards to reducing greenhouse gas emissions , without negatively impacting the economic competitiveness of the company or its ability to effectively provide postal services .
Modification of procedures	7	[Company] introduced the [programme] to promote low - carbon practices that minimize carbon dioxide emissions by reducing power consumption when computers are not in use .
Modification of procedures	12	In fiscal 2014 this system produced an amount of recycled paper equivalent to 496 trees , and in calendar 2014 contributed to a reduction in greenhouse gas emissions of approximately 52.2 metric tons .
Modification of procedures	7	In fiscal year 2015 , [Company] once again promoted efforts in line with [location]'s [campaign] and [campaign] campaigns , official efforts to reduce electric power consumption by limiting the use of air conditioning in summer and winter through measures such as the introduction of more relaxed office dress codes .

Table ST1: **Example of common initiatives** The table shows a few examples of sustainability initiatives. Activity classified into SDG 7 and 12 are similar in scope. However, the algorithm seem to assign SDG 7 to activities that are more closely related to renewable energy and SDG 12 to activities that are more closely related to efficient use of resources. While reporting the examples, we have replaced the company names with "[Company]" in order to shift the focus from the specific companies on their language. Similarly we have renamed policies, location, campaigns and programmes.

Below we provide examples of initiatives excluded from our analysis because they are not directly related to GHG emissions

Activity	SDG	Text
modification of procedures	6	In 2017 , in addition to daily activities such as raising employees' awareness of saving water and conducting patrols to check water leakage , efforts were made to raise the ratio of utilization of recycled water at the sites where the wastewater recycling system has been introduced .
asset modification	7	Much of that build – including vastly improved wireless coverage in the [location] corridor between [location] and [location] – will provide benefits to [population] for generations to come ..For instance , for the first time in [location] , [company] installed a solar - powered cell site as part of its Olympic - grade wireless network ..This site will continue to provide cellular service to thousands of residents in [location] , a growing community north of [location] ..For each of the 42 new cell sites we built , we consulted with local governments and [population] , conducting environmental impact studies that included frog mating and migratory bird studies , as well as locating [population] burial grounds ..One of the advantages of an IP network is that it does n't need as much cabling and other infrastructure as older technologies do ..As a result , we reduced the amount of materiel we used
communications	11	To foster a positive corporate image and increase exchanges with local communities , the company has promoted plant tours , and the number of people annually participating in such tours has surpassed 30,000 ..In addition , the company organized cleanup campaigns with respect to sidewalks and roads near its production plant
new products	12	[company] also developed an EE - flute corrugated sheet strong enough to replace the non - recyclable polystyrene core in foam board display sheets .
association	13	[Company] partnered with the [organisation] , and our supplier , [company] , to provide needed climate information by piloting the installation of weather stations that provide more accurate forecasts via SMS messages to 500 cocoa smallholder farmers .
r&d investments	14	In 2014 we supported a stakeholder engagement and research initiative involving commercial fishers , government regulators , research organizations and other operators as part of our [location] field development .In addition to building strong stakeholder relationships , the group aims to contribute to the broader scientific understanding of fish distributions and stock structures which will assist long - term sustainable fisheries management as well as help our ongoing efforts to understand and mitigate the risks of our activities .
donation & funding	15	In [location] , we 're protecting more than 3600 acres of pine and hardwood forest in [location] , along the southern coast of [location] .

C.1 Sustainability behaviour and renewable energy sources

Here we investigate the relationship between sustainability behaviour and energy sources in companies' power generation. We focus on a small sample of companies for which we could collect detail information on energy mix from TruCost. The summary statistics of the sample is shown in table ST2. The top table shows the assets characteristics and the total number of firms and countries. The table shows that the sample comprises only a few firms, by they are high emitters and have a large value of invested capital. The bottom table shows the average power generated segmented by energy source in (GWh). Figure S2 shows the distribution of the differences between the power generated by renewable energy sources and the power generated by fossil fuels as a function of time. The figure illustrates that renewable sources have become significantly more important in recent years.

	Size	Invested capital	Tangibility	Emissions (tCO_2e)	Emissions (cumulative)	# of firms	# of countries
2012	9.20	39189.0	0.57	52534147	2.626707e+09	50	20
2013	8.99	31345.0	0.56	49026495	5.862456e+09	66	23
2014	8.59	27609.0	0.57	41308566	8.919290e+09	74	24
2015	8.66	26740.0	0.59	39351176	1.238219e+10	88	28
2016	8.40	22626.0	0.58	37482783	1.639285e+10	107	28
2017	8.38	24509.0	0.57	36454947	2.069453e+10	118	28
2018	8.29	26623.0	0.54	33730489	2.531561e+10	137	31
2019	8.03	25042.0	0.56	29585256	3.034511e+10	170	31
2020	7.95	25465.0	0.56	23251516	3.466989e+10	186	33
Summary	8.50	27683.1	0.56	38080597	1.746763e+10	228	35

	Biomass	Coal	Geothermal	Hydroelectric	LNG	LandFillGas	NaturalGas	Nuclear	Oil	Solar	WaveTidal	Wind
2012	460.573200	32413.149200	120.473000	11077.176400	0.000000	2.804400	21587.342400	24803.831400	2544.166600	92.290200	8.006000	2663.342200
2013	582.176667	29953.131212	513.111212	11899.041364	3820.011364	3.954091	18031.704394	19211.701667	2479.524848	4637.046970	5.454545	2728.745909
2014	445.327432	25089.408919	359.959459	11460.500541	2796.199865	3.495811	13295.817703	17426.842568	1775.940676	278.719459	3.645676	2329.009595
2015	435.545114	23934.817500	377.758750	9442.388864	1242.747500	3.300341	14767.924091	14733.679205	1220.081477	272.010227	5.255227	2554.686591
2016	386.071963	23973.640654	268.457850	7828.139533	1001.784953	2.312430	12310.810561	13300.829720	1066.763832	275.066916	1.335421	2445.860935
2017	330.859322	19474.034237	275.392034	7472.469407	1022.136271	8.538305	13989.516271	12334.593814	836.979661	269.480593	1.244576	2366.162034
2018	611.187664	16308.269708	226.106496	6391.815474	897.712190	7.592555	15330.212336	9775.431533	731.609562	312.094015	1.174161	2354.474307
2019	545.020824	13871.658235	186.303412	5724.567882	709.106235	7.061941	11914.334353	7814.781118	434.534471	407.497059	0.803882	3280.106059
2020	474.543876	10364.738074	193.077258	6038.912608	303.399086	2.184086	10087.608640	6961.227097	292.801344	584.103726	2.918548	3348.176645

Table ST2: **Average statistics of the sample** The top table shows the summary statistics of the asset characteristics of firms in the sample. The bottom table shows the sources in the energy mix in GWh.

The goal of this analysis is to estimate the relationship between the behaviour most prevalent in companies with emission pathways aligned with the targets of the Paris Agreement and the relative abundance of renewable sources in the energy mix, $\mathcal{R}$ (the y-axis in figure S2). Therefore, we run the following regression:

$$\mathcal{R}_{i,t} = \alpha + \beta \mathcal{I}_{E,i,t} + \sum_j \gamma_j \mathcal{X}_{j,i,t} + \boldsymbol{\delta} \mathbf{C}_i + \boldsymbol{\eta} \mathbf{S}_i + \boldsymbol{\mu} \mathbf{R} + \epsilon_{i,t} \qquad (1)$$

Where $\mathcal{I}_{E,i,t}$ is the total number of initiatives in negative and positive excess effort for company i in year t where $t \leq T$, the year in which the alignment is calculated. The control

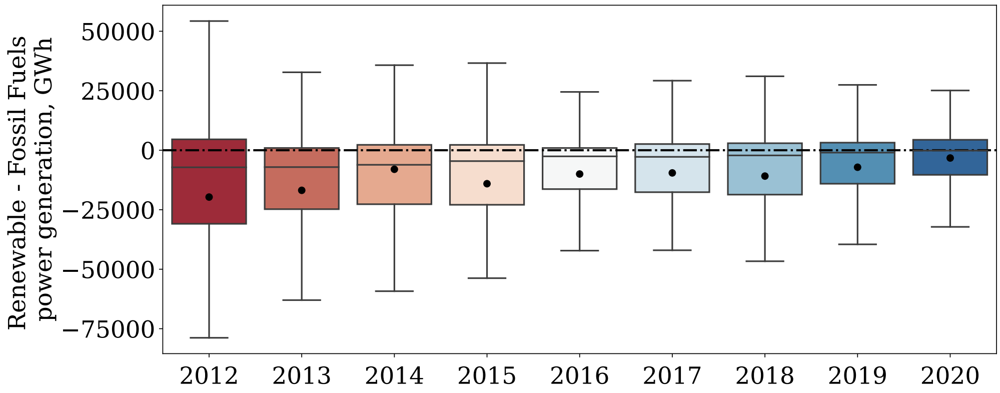

Fig. S2 | Relative abundance of renewable sources. Temporal evolution of the difference between the power produced by renewable sources and the power generated by fossil fuels before matching with any other dataset (N = 1513). The line of the box plots are median lines, the solid circles are means, the edges of the boxes are the quartile range: the 25th and 75th percentile.

set **X** includes Revenue (log), Total invested capital (log), and proportion of tangible assets (Tangibility). We also include country (**C**), sector (**S**), and report characteristics (**R**) fixed effects. Because most of the firms are concentrated in the Utilities sectors, to avoid unbalance the dataset we used the GICS Industry group as opposed to GICS Sector classification to allow for more variability in the fixed effects. The results of the regression for the below 2°C and the well below 2°C targets and the year of estimation of alignment are shown in table ST3. The table shows that the initiatives in positive excess effort are positive and strongly statistically significantly associated with the relative abundance of renewable sources in the total power generated by the companies in our sample.

	Below 2°C		Well Below 2°C	
	Negative excess effort	Positive excess effort	Negative excess effort	Positive excess effort
2018	-0.02	0.05***	-0.01	0.05***
2019	-0.0	0.04***	-0.0	0.04***
2020	0.0	0.04***	-0.01	0.04***

Table ST3: **Sustainability behaviour and renewable sources.** The table shows that initiatives in positive excess effort are positively correlated with the relative power generated by renewable sources.

[1] Hirlea, D., Bryant, C., Zollo, M. & Rei, M. Contextual sentence classification: Detecting sustainability initiatives in company reports. Available at https://arxiv.org/abs/2110.03727 (2021).

[2] Devlin, J., Chang, M.-W., Lee, K. & Toutanova, K. Bert: Pre-training of deep bidirectional transformers for language understanding. In *Proceedings of the 2019 Conference of the North American Chapter of the Association for Computational Linguistics: Human Language Technologies, Volume 1 (Long and Short Papers)*, 4171–4186 (2019).

[3] Liu, Y. *et al.* RoBERTa: A robustly optimized bert pretraining approach. *arXiv preprint arXiv:1907.11692* (2019).

[4] Paszke, A. *et al.* Pytorch: An imperative style, high-performance deep learning library. *Advances in neural information processing systems* **32** (2019).

[5] Wolf, T. *et al.* Huggingface's transformers: State-of-the-art natural language processing. *arXiv preprint arXiv:1910.03771* (2019).

D Supplementary figures

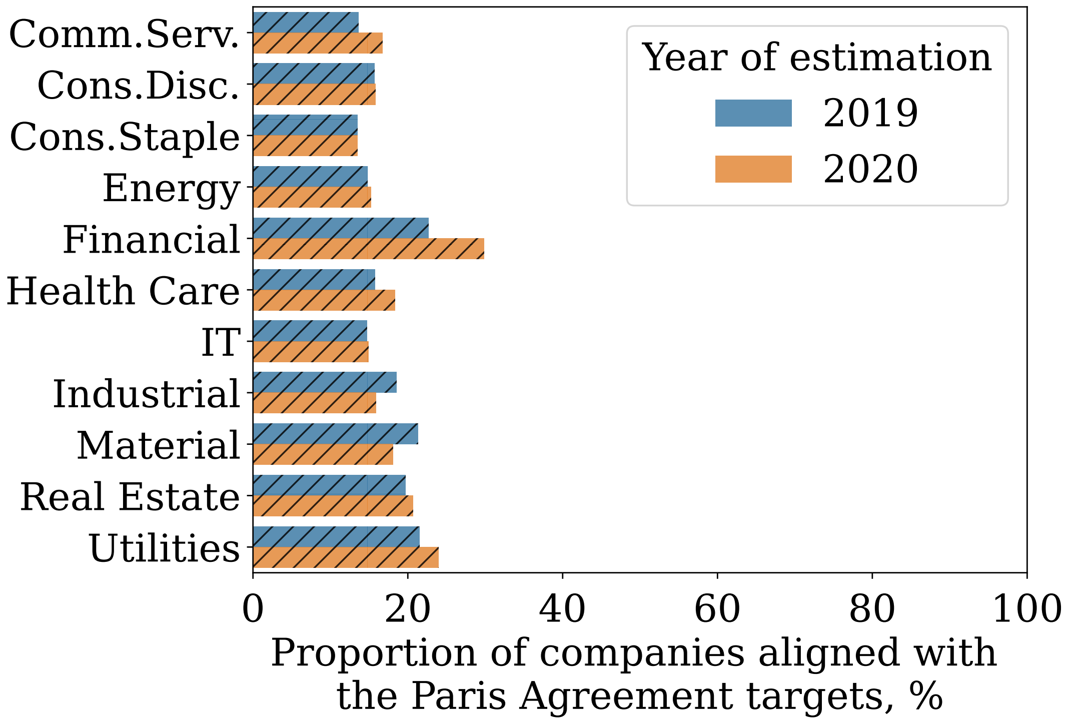

Fig. S3 | Alignment with Paris agreement targets. The figure shows the fraction of companies in the TruCost database, by sectors, with emissions pathways aligned with the target set by the Paris agreement of limit temperature increase well below 2°C. For illustrative purposes, we show alignment calculated in 2019 and 2020 for all companies with available data in both years (12606). If we look at the two years separately, we have 12700 companies in 2019 and an average of 18% of them are aligned with the Paris targets. In 2020 we have 13600 companies and an average of 19% of them are aligned. Data are from TruCost which estimates emissions pathways using the SDA (GEVA) approach, for high (low)-emitting companies with homogeneous (heterogeneous) business activities.

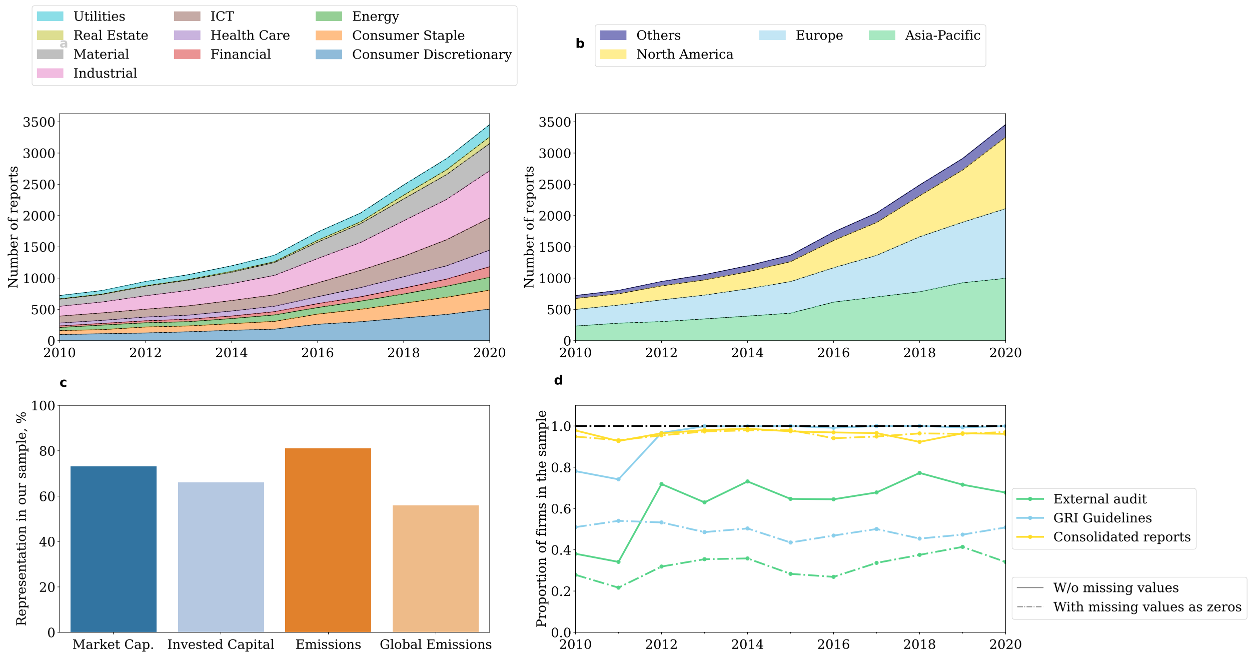

Fig. S4 | Overview of of our sample. Panel **a** shows the total number of reports per year and sector in our sample. Panel **b** shows the total number of reports by macro region. Panel **c** shows that companies in our sample cover $\sim 70\%$ of global market capitalisation; $\sim 60\%$ of total invested capital; $\sim 80\%$ of the direct control and first tier emissions covered by TruCost, and $\sim 50\%$ of global emissions (both business and non-business emissions). Panel **d** shows some characteristics of the reports. Continuous lines in the panel denote the average incidence of a report characteristic after removing missing values from the Asset4 database. The dotted lines show the average incidence after replacing missing values with zeros.

	Size	Invested capital	Tangibility	Emissions (tCO_2e)	Emissions (cumulative)	# of firms	# of countries
2010	8.97	28611.0	0.32	8459956	6.099629e+09	721	40
2011	8.93	26447.0	0.32	8745757	1.311373e+10	802	39
2012	8.82	26372.0	0.32	8494030	2.114058e+10	945	39
2013	8.68	24347.0	0.31	8066904	2.965117e+10	1055	41
2014	8.53	24586.0	0.31	7172673	3.822968e+10	1196	45
2015	8.32	21224.0	0.31	6657137	4.732333e+10	1366	44
2016	8.10	18880.0	0.31	6329543	5.831142e+10	1736	45
2017	8.02	18420.0	0.30	6084758	7.073041e+10	2041	48
2018	7.86	16879.0	0.29	5496781	8.441190e+10	2489	47
2019	7.66	15213.0	0.32	4985430	9.892947e+10	2912	46
2020	7.37	15512.0	0.30	3664333	1.115934e+11	3456	49
Summary	8.30	21499.2	0.30	6741572	5.268498e+10	4191	51

Table ST4: **Summary statistics of the population**. The table shows the summary statistics of the companies in our sample. The first four columns are average values across the sample. The decrease in the average emissions is due to the inclusion of a large number of smaller companies in the TruCost database on a year-to-year basis. Indeed, average size and invested capital have also declined. The bottom row shows average values as well as total number of unique entities and countries

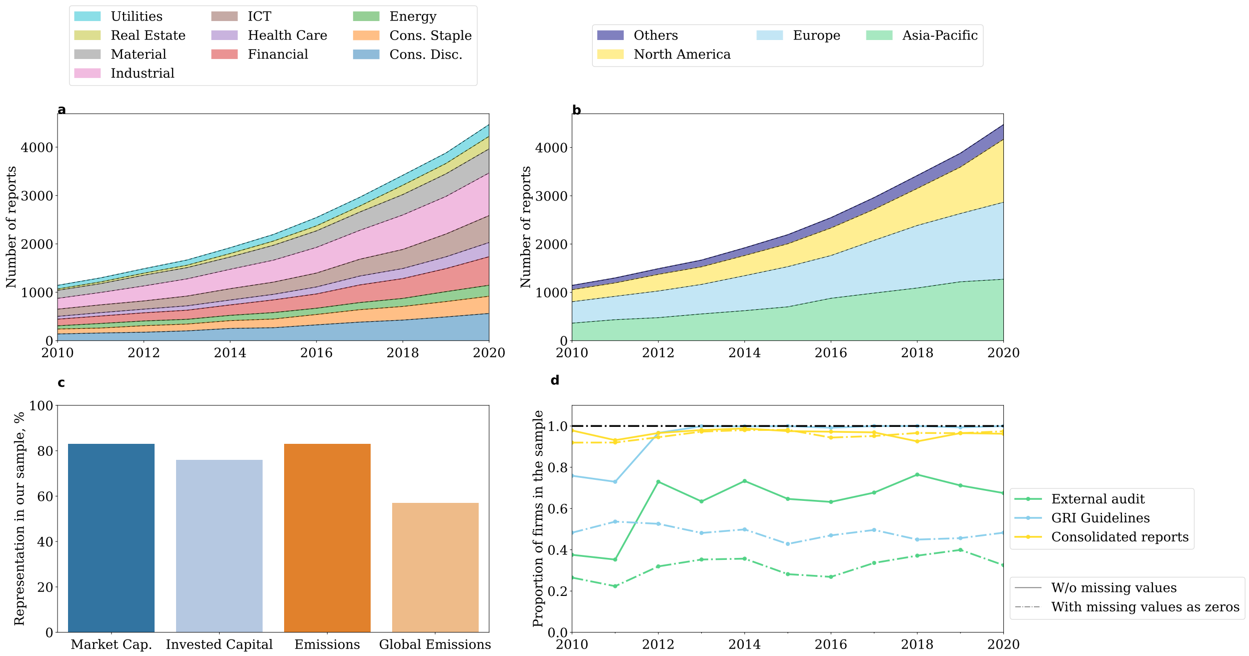

Fig. S5 | Overview of the full population. The figure shows the descriptive statistics of the full population before matching the behavioural dataset with accounting and emission data. Panel **a** shows the total number of reports per year and sector. Panel **b** shows the total number of reports by macro region. Note, the total number of companies analysed in the main text is lower than the total number companies in our behavioural dataset as not all companies have the emission and accounting information used in the analysis. Panel **c** shows that companies in the full population cover $\sim 80\%$ of global market capitalisation; $\sim 70\%$ of total invested capital; $\sim 80\%$ of the direct control and first tier emissions covered by TruCost, and $\sim 60\%$ of global emissions (both business and non-business emissions). Panel **d** shows some characteristics of the reports. Continuous lines in the panel denote the average incidence of a report characteristic after removing missing values from the Asset4 database. The dotted lines show the average incidence after replacing missing values with zeros.

	SDG 12	SDG 7	SDG 15	SDG 6	SDG 11	SDG 14	SDG 13	Total
donation & funding	5404	2355	9239	2959	9740	336	99	30132
modification of procedures	19679	3446	1629	2594	301	99	35	27783
asset modification	6987	13804	979	4601	763	51	15	27200
assessment and measurement	9892	1542	2536	2395	386	166	138	17055
communication	8959	1688	2616	1288	795	125	219	15690
association	6069	1412	2819	1077	704	145	167	12393
volunteerism	1386	146	3369	399	2146	155	3	7604
r&d investments	2945	2301	844	763	152	77	33	7115
new products	4054	2257	195	392	172	27	8	7105
training	4697	493	714	399	454	50	47	6854
incentives	1472	691	81	76	35	0	5	2360
organizational structuring	1518	229	134	100	161	7	11	2160
adoption of standards and rules	628	85	83	62	8	3	14	883
pricing	24	171	2	22	11	0	0	230
Total	73714	30620	25240	17127	15828	1241	794	164564

Fig. S6 | Behavioural matrix. The figure shows the full behavioural matrix of the companies in our sample.

SDG 7	SDG 12	SDG 15	SDG 6	SDG 11	SDG 13	SDG 14	Total	
11237	4202	142	996	165	15	9	16766	asset modification
2970	5475	202	518	41	34	8	9248	modification of procedures
1315	2664	231	412	82	125	16	4845	assessment and measurement
1575	843	1134	236	324	94	27	4233	donation & funding
1387	1904	331	170	53	193	9	4047	communication
1605	1617	28	87	20	8	0	3365	new products
1047	1591	372	139	42	147	20	3358	association
1568	1313	149	141	36	26	5	3238	r&d investments
392	826	61	48	25	42	5	1399	training
521	423	11	22	6	4	0	987	incentives
93	201	290	45	65	3	19	716	volunteerism
193	273	15	7	15	10	0	513	organizational structuring
71	164	12	7	1	12	0	267	adoption of standards and rules
138	4	0	5	1	0	0	148	pricing
24112	21500	2978	2833	876	713	118	53130	Total

Fig. S7 | GHG initiatives in the behavioural matrix. The figure shows the full behavioural matrix of the companies in our sample. Differently from figure S6, here we show only the initiatives that refer directly to an activity that is intended to reduce GHG emissions. Initiatives related to other environmental issues have been excluded from this analysis

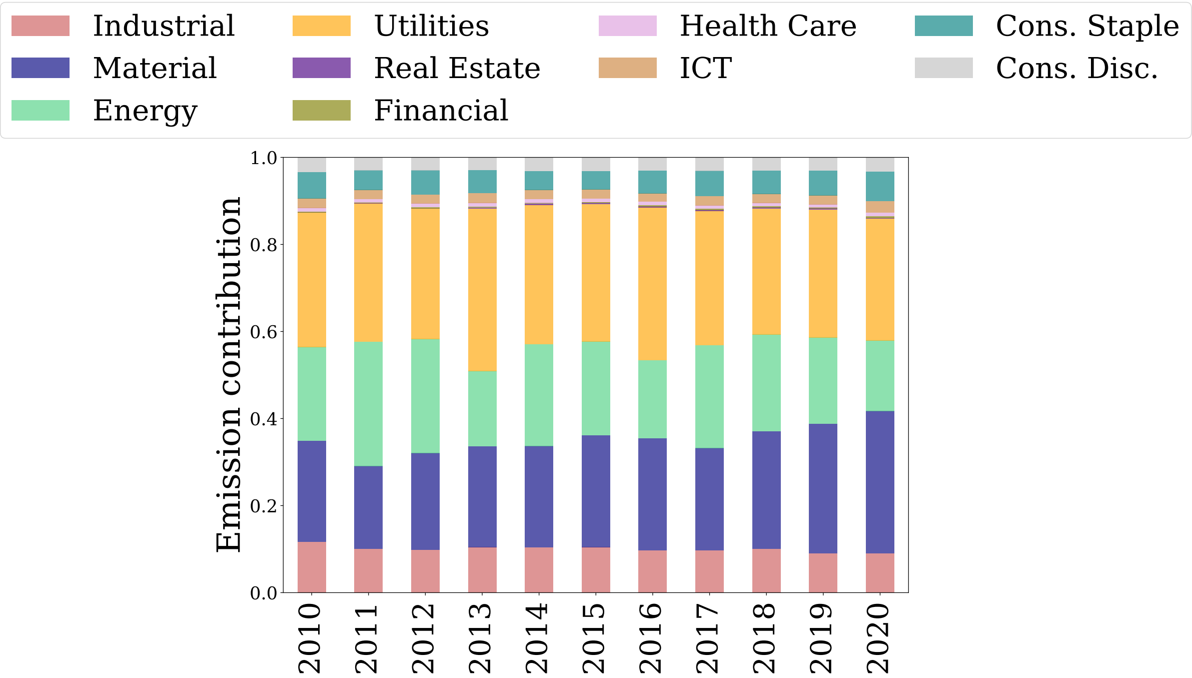

Fig. S8 | Contribution to total emissions by sectors. The figure shows that the Material, Industrial, Utilities and Energy sectors account for $\sim 90\%$ of the emissions in our sample

	SDG 7	SDG 12	SDG 15	SDG 6	SDG 11	SDG 13	SDG 14	Total
asset modification	4982	2555	95	505	110	5	7	8259
modification of procedures	1166	2137	113	244	25	18	3	3706
donation & funding	1158	417	691	129	211	47	19	2672
assessment and measurement	588	1238	147	174	48	63	12	2270
r&d investments	1097	894	101	105	27	17	4	2245
communication	901	819	172	92	33	88	1	2106
association	583	784	200	69	35	82	10	1763
new products	903	725	18	47	15	1	0	1709
training	240	398	33	24	20	32	5	752
incentives	415	171	7	16	3	2	0	614
volunteerism	57	93	142	21	36	0	8	357
organizational structuring	82	122	9	3	11	3	0	230
pricing	133	4	0	5	1	0	0	143
adoption of standards and rules	29	75	6	4	0	4	0	118
Total	12334	10432	1734	1438	575	362	69	26944

Fig. S9 | Behavioural matrix in the Material, Industrial, Utilities and Energy sectors. The figure shows the full behavioural matrix of the companies in the sectors (including only GHG related initiatives and after matching with the other datasets).

Hard to abate	Size	Invested capital	Tangibility	Emissions (tCO_2e)	Emissions (cumulative)	# of firms	# of countries
2010	8.93	19417.0	0.39	14313779	5.324726e+09	372	38
2011	8.89	19821.0	0.39	14753278	1.159487e+10	425	36
2012	8.79	19281.0	0.39	14077617	1.867591e+10	503	36
2013	8.60	17879.0	0.38	13433691	2.618534e+10	559	38
2014	8.50	18041.0	0.39	12283787	3.382586e+10	622	41
2015	8.21	15331.0	0.40	11239719	4.194094e+10	722	42
2016	8.00	13561.0	0.39	10937334	5.166423e+10	889	44
2017	7.97	13490.0	0.39	10703145	6.254932e+10	1017	43
2018	7.86	12828.0	0.38	9826579	7.461636e+10	1228	43
2019	7.66	11762.0	0.40	9131469	8.738216e+10	1398	44
2020	7.39	11461.0	0.39	6817485	9.825605e+10	1595	42
Summary	8.30	15715.6	0.40	11592534	4.654689e+10	1951	45

Table ST5: **Summary statistics of companies in thee high emissions sectors**. The table shows the summary statistics of the companies in th Energy, Utilities, Material and Industrial sectors. The first four columns are average values across the sample. The decrease in the average emissions is due to the inclusion of a large number of smaller companies in the TruCost database on a year-to-year basis. Indeed, average size and invested capital have also declined. The bottom row shows average values as well as total number of unique entities and countries. Comparing the table with table ST4 we observe a significantly larger presence of tangible assets and substantially larger average emissions.

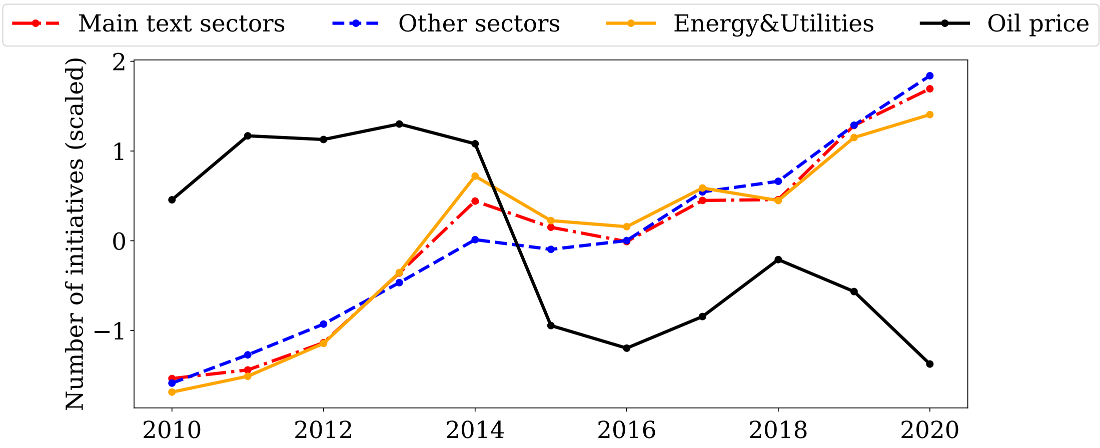

Fig. S10 | Number of initiatives and oil price. The figure shows the total number of initiatives (scaled to zero mean and unitary variance) in the four most polluting sectors (red), the other sectors (blue), and Energy and Utilities (orange). The black line shows the oil price during the observation period (scaled). Overall, the figure shows that the drop in number of initiatives in 2014-2016 is more pronounced in the sectors analysed in the main text and coincided with a drastic drop in oil prices (indeed the effect is stronger in the Energy and Utilities sectors).

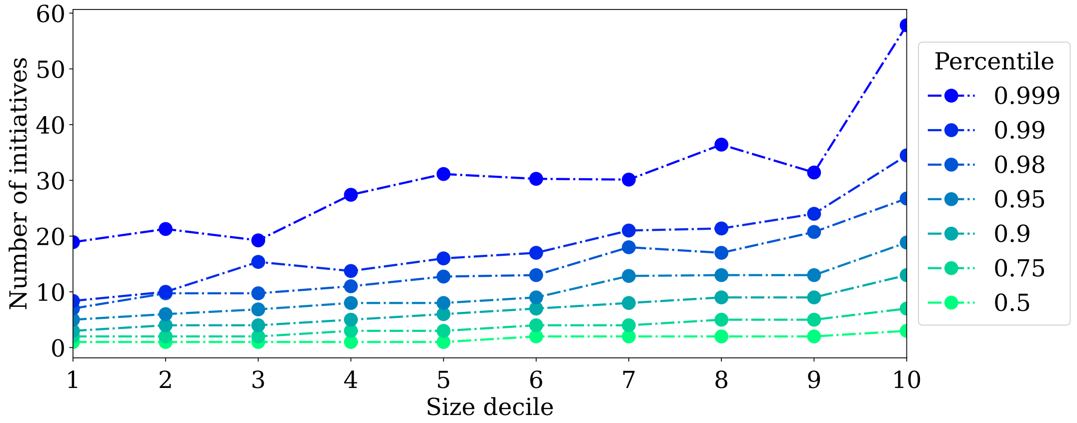

Fig. S11 | Number of initiatives and companies' size. The x-axis is the size decile. The y-axis is the number of initiatives in the percentile coded by the colour map. The figure shows that there is a significant heterogeneity in the number of initiatives that different corporations undertake, and that this heterogeneity is more evident for larger companies.

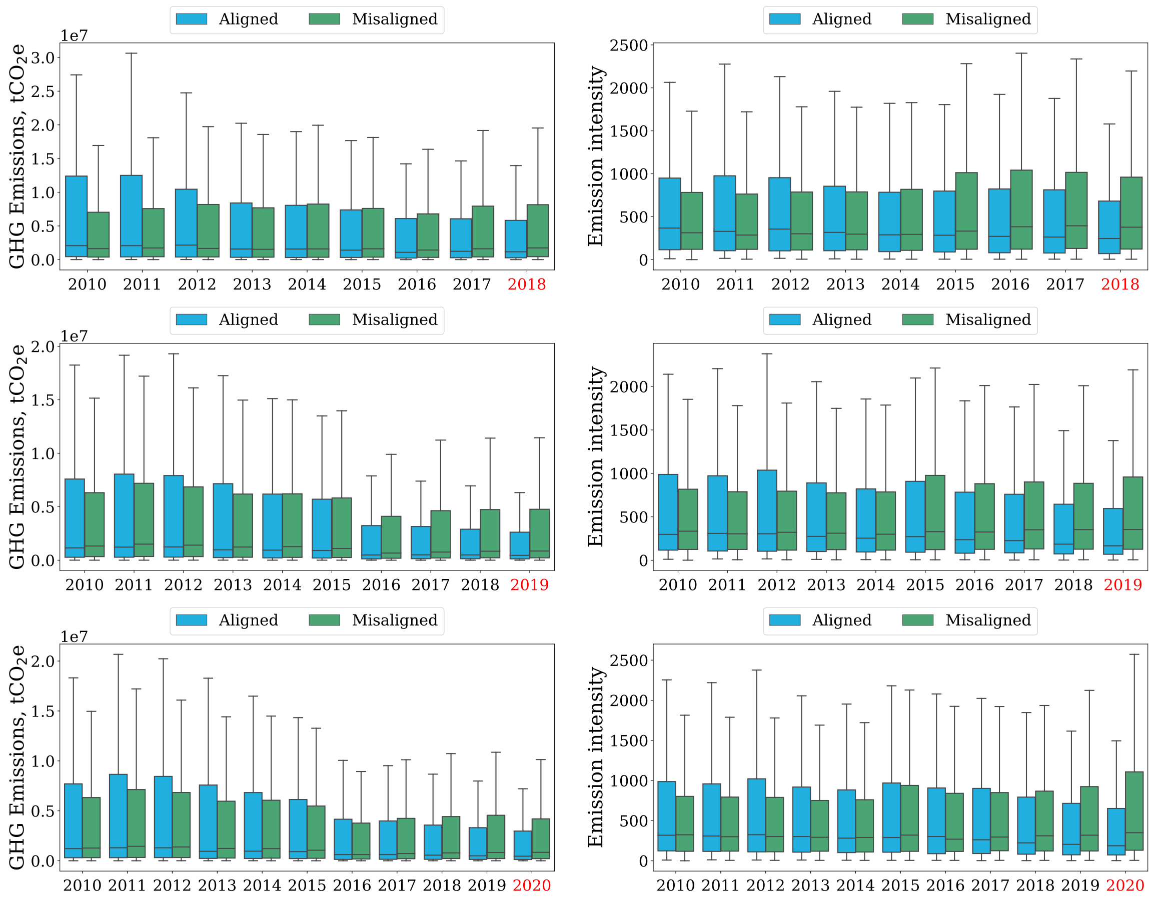

Fig. S12 | Emissions in the full sample (Climate targets). The figure shows the absolute emissions (left panel) and emission intensity (right panel) of the aligned and misaligned populations. The figure includes all companies in the TruCost database. The line of the box plots are median lines, the edges of the boxes are the quartile range: the 25th and 75th percentile. Overall, companies with emission pathways aligned with the targets set by the Paris agreement as of 2018,2019,2020 (red marks) have decreased their emissions during the observation period. Emission intensity is calculated as total emissions over sales (in USD)

Behavioral differences

	SDG 7	SDG 15	SDG 13	SDG 11	SDG 14	SDG 6	SDG 12	Total
r&d investments	2.4	0.2	0.1	0	0	-0.3	-0.5	1.9
new products	1.8	-0.1	0	0	0	-0.1	0.1	1.7
association	0.9	0.3	-0.1	0.2	0	0	-0.2	1.1
communication	0.6	0.1	0.7	-0.1	0	0	-0.4	0.9
incentives	0.7	0	0	0	0	-0.1	0.1	0.7
donation & funding	1.1	0.5	-0.1	0	-0.1	-0.3	-0.6	0.5
pricing	0.6	0	0	0	0	-0.1	0	0.5
adoption of standards and rules	0	0	0	0	0	0	0	0
organizational structuring	0.1	0	0	-0.1	0	0	-0.1	-0.1
volunteerism	-0.2	0	0	0.1	-0.1	-0.1	-0.1	-0.4
training	-0.1	0.1	0	-0.2	0	-0.1	-0.4	-0.7
assessment and measurement	-0.6	0.3	0	-0.1	-0.1	-0.1	-1	-1.6
modification of procedures	0.3	0.2	0	0.1	0	-0.8	-1.5	-1.7
asset modification	0.6	-0.2	0	0.3	0	-0.4	-3	-2.7
Total	8.2	1.4	0.6	0.2	-0.3	-2.4	-7.6	0

Fig. S13 | Difference behavioural matrix in the climate targets analysis. The figure shows the difference behavioural matrix in the aligned companies and misaligned companies. The matrix refers to the population analysed in figure 4 in the main text, which comprises companies in the largest size quartile of our sample.

Sector disclosure	Country disclosure	Profitability	Size	Investment	Tangibility
0.22***	0.28***	0.05***	0.33***	0.35***	0.05***

Table ST6: **Heckman correction.** The table shows the coefficients from the Probit model in the first step of the Heckman's correction. The dependent variable is an indicator that takes the value of one if company i publish a sustainability report in year y. Information on issuance is from Refinitiv Asset4. *,**,*** denote statistical significance at the 10%,5%, and 1% level, respectively, calculated from the two-tailed p-values for the t-statistics of the parameters.

	Magnitude of deviation		Probability of alignment	
	Negative excess effort	Positive excess effort	Negative excess effort	Positive excess effort
Model 1	0.0	0.01	0.01	0.07
Model 2	-0.0	-0.01*	0.01	0.07
Model 3	-0.0	-0.01*	0.03	0.1*
Model 4	-0.0	-0.01*	0.03	0.1*
Model 5	-0.0	-0.01*	0.03	0.1*

	Magnitude of deviation		Probability of alignment	
	Negative excess effort	Positive excess effort	Negative excess effort	Positive excess effort
Model 1	0.01	-0.0	0.01	0.07
Model 2	-0.0	-0.02***	0.01	0.08
Model 3	-0.0	-0.02***	0.03	0.1*
Model 4	-0.0	-0.02***	0.03	0.1*
Model 5	-0.0	-0.02***	0.03	0.1*

Table ST7: **The importance of the what and the why of corporate sustainability behaviour.** The table shows that the results of our main analysis (figure 4 in the main text) are weaker when we exclude the SDG dimensions (top) and the activity types (bottom) from the construction of the differential behaviour variables. *,**,*** denote statistical significance at the 10%,5%, and 1% level, respectively, calculated from the two-tailed p-values for the t-statistics of the parameters.

	Magnitude of deviation		Probability of alignment	
	Negative excess effort	Positive excess effort	Negative excess effort	Positive excess effort
Model 1	0.01	0.0	-0.0	0.1**
Model 2	0.0	-0.02***	-0.0	0.11**
Model 3	0.0	-0.03***	0.01	0.13**
Model 4	0.0	-0.03***	0.01	0.13**
Model 5	0.0	-0.03***	0.01	0.13**

Table ST8: **Alternative control for reports characteristics** The table shows that the results of our main analysis (figure 4 in the main text) are left qualitatively unchanged when we use an indicator variable as opposed to fixed effects to control for report characteristics. When using an indicator variable, we assume that missing data correspond to the absence of a specific report characteristic. *,**,*** denote statistical significance at the 10%,5%, and 1% level, respectively, calculated from the two-tailed p-values for the t-statistics of the parameters.

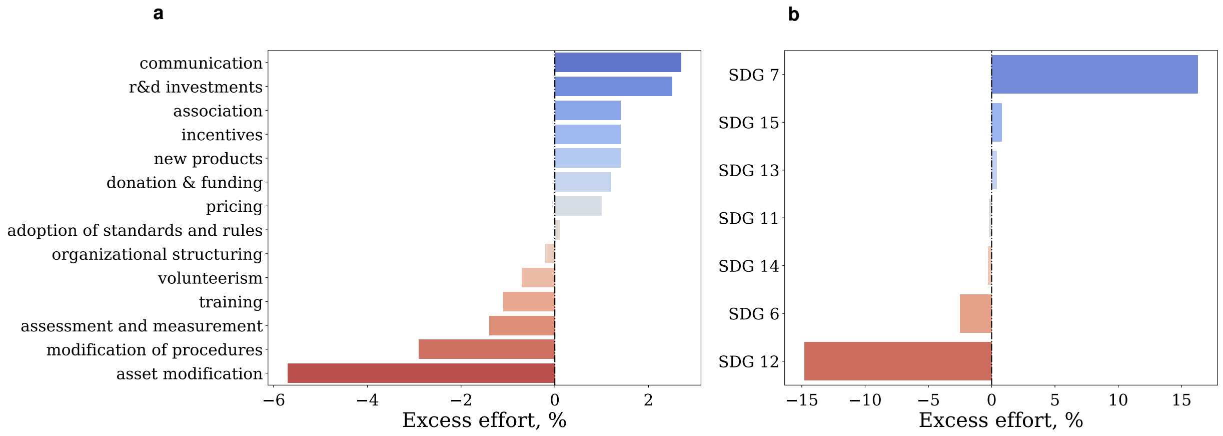

c

	Magnitude of deviation		Probability of alignment	
	Negative excess effort	Positive excess effort	Negative excess effort	Positive excess effort
Model 1	0.02**	0.01	-0.03	0.13**
Model 2	0.01	-0.02***	-0.03	0.13**
Model 3	0.01	-0.02***	-0.02	0.14**
Model 4	0.01	-0.02***	-0.02	0.15***
Model 5	0.01	-0.02***	-0.02	0.15***

Fig. S14 Differential behaviour can explain alignment with climate targets (well below 2°C target). Panel **a** and **b** show the excess sustainability effort of companies with emission pathways aligned with the climate targets. The excess effort is the difference in the relative incidence of activities and SDG in the two populations. Blue (red) bars indicate activities and SDGs that are more prevalent in the aligned (misaligned) population. Panel **c** shows the estimation of the association of the total number of initiatives in negative (red) and positive (blue) excess effort with the magnitude of the deviation and the probability of alignment. The model number on the leftmost column in the table correspond to the incremental addition of control factors as in figure 3 panel **d**. In each model, we control for fixed effects, source of emission data, self-selectivity, and we adjust standard errors for heteroskedasticity. *,**,*** denote statistical significance at the 10%,5%, and 1% level, respectively, calculated from the two-tailed p-values for the t-statistics of the parameters. Overall, the figure shows that results are robust when we use a more stringent target as dependent variable in our analysis.

	Magnitude of deviation		Probability of alignment	
Below 2°C<br>$Size_q$	Negative excess effort	Positive excess effort	Negative excess effort	Positive excess effort
0	0.0	-0.02***	0.01	0.13**
1	-0.01	0.01	0.07	0.05
2	0.01	-0.02***	0.0	0.13**
3	0.0	-0.02**	0.01	0.11**
4	0.0	-0.03***	0.02	0.13**

	Magnitude of deviation		Probability of alignment	
Below 2°C<br>Year	Negative excess effort	Positive excess effort	Negative excess effort	Positive excess effort
2018	-0.01	-0.02**	0.05	0.08
2019	0.0	-0.02***	0.01	0.13**
2020	-0.01*	-0.03***	-0.01	0.11**

Table ST9: **Robustness of the effect across size quartiles and estimation windows.** The table shows the estimation of the model for different size quartiles and time windows. Size quartile equal to zero in the first row correspond to the full population. The effects in the different estimation windows are estimated over the full populations. *,**,*** denote statistical significance at the 10%,5%, and 1% level, respectively, calculated from the two-tailed p-values for the t-statistics of the parameters. Overall, the table shows that results are robust to different subsample of our population and across different time windows.

	Full	Q_1	Q_2	Q_3	Q_4	Average		2018	2019	2020	Average
donation & funding	0.9	4.2	0.9	0.4	0.5	1.50	incentives	3.1	0.6	2.3	2.00
r&d investments	1.3	0.1	0.8	1.5	1.9	1.08	donation & funding	1.3	0.9	2.5	1.57
new products	1.1	-0.3	-0.5	2.2	1.7	0.77	communication	1.4	0.6	2.1	1.37
communication	0.6	0.6	1.0	0.5	0.9	0.75	new products	0.8	1.1	0.6	0.83
incentives	0.6	-0.3	1.7	-0.1	0.7	0.50	r&d investments	0.2	1.3	0.4	0.63
association	0.5	-0.2	-0.9	1.0	1.1	0.25	pricing	0.8	0.3	0.5	0.53
pricing	0.3	0.2	0.1	0.0	0.5	0.20	association	0.2	0.5	0.7	0.47
adoption of standards and rules	0.0	0.5	-0.2	-0.1	0.0	0.05	adoption of standards and rules	0.0	0.0	-0.1	-0.03
organizational structuring	-0.0	-0.4	0.5	0.1	-0.1	0.02	organizational structuring	-0.3	-0.0	-0.3	-0.20
volunteerism	-0.1	-0.1	0.1	0.1	-0.4	-0.08	training	-0.3	-0.2	-0.3	-0.27
training	-0.2	-0.2	0.2	0.1	-0.7	-0.15	volunteerism	-0.4	-0.1	-0.4	-0.30
modification of procedures	-0.7	-0.9	2.7	-1.6	-1.7	-0.38	assessment and measurement	-1.7	-1.0	-1.2	-1.30
assessment and measurement	-1.0	-1.4	-1.3	-0.3	-1.6	-1.15	modification of procedures	-2.7	-0.7	-1.4	-1.60
asset modification	-3.5	-0.7	-5.1	-3.1	-2.7	-2.90	asset modification	-2.1	-3.5	-6.4	-4.00

	Full	Q_1	Q_2	Q_3	Q_4	Average		2018	2019	2020	Average
SDG 7	4.8	-0.0	0.8	5.6	8.2	3.88	SDG 7	9.9	4.8	7.2	7.30
SDG 15	1.2	4.4	2.3	-1.0	1.4	1.66	SDG 11	-0.3	-0.3	0.3	-0.10
SDG 13	-0.1	0.8	-0.7	-0.4	0.6	0.04	SDG 13	-0.3	-0.1	-0.1	-0.17
SDG 11	-0.3	0.1	-0.5	-0.2	0.2	-0.14	SDG 14	-0.1	-0.3	-0.2	-0.20
SDG 6	-0.6	0.0	1.7	0.2	-2.4	-0.22	SDG 6	0.1	-0.6	-0.4	-0.30
SDG 14	-0.3	-0.3	-0.2	-0.1	-0.3	-0.24	SDG 15	-1.4	1.2	-0.7	-0.30
SDG 12	-4.9	-3.9	-3.4	-3.4	-7.6	-4.64	SDG 12	-7.6	-4.9	-7.1	-6.53

Table ST10: **Excess effort across size and estimation windows.** These are the numerical value associated with table 5 in the main text. Positive values denote actions and SDGs more prevalent in the aligned population (blue circles in table 5 in the main text).

well Below 2°C Size$_q$	Magnitude of deviation Negative excess effort	Positive excess effort	Probability of alignment Negative excess effort	Positive excess effort
0	0.01	-0.02***	-0.02	0.15***
1	0.01**	-0.01**	-0.02	0.1*
2	0.01**	-0.01**	-0.04	0.13**
3	-0.0	-0.03***	-0.01	0.12**
4	0.01	-0.02***	-0.02	0.15***

well Below 2°C Year	Magnitude of deviation Negative excess effort	Positive excess effort	Probability of alignment Negative excess effort	Positive excess effort
2018	-0.0	-0.01	-0.01	0.1
2019	0.01	-0.02***	-0.02	0.15***
2020	-0.01*	-0.02***	-0.06	0.07

Table ST11: **Robustness of the effect across size quartiles and estimation windows (well below 2°).** The table shows the estimation of the model for different size quartiles and time windows. Size quartile equal to zero in the first row correspond to the full population. The effects in the different estimation windows are estimated over the full populations. *,**,*** denote statistical significance at the 10%,5%, and 1% level, respectively, calculated from the two-tailed p-values for the t-statistics of the parameters. Overall, the table shows that results are robust even when we use a more stringent climate target.

	Full	Q_1	Q_2	Q_3	Q_4	Average		2018	2019	2020	Average
donation & funding	1.2	4.3	0.6	0.3	1.2	1.60	donation & funding	2.2	1.2	1.8	1.73
r&d investments	1.8	-0.5	-1.1	3.1	2.5	1.00	incentives	2.8	0.8	1.3	1.63
incentives	0.8	0.5	0.4	0.8	1.4	0.77	communication	1.7	1.3	1.4	1.47
communication	1.3	-1.8	0.1	2.0	2.7	0.75	r&d investments	1.2	1.8	0.4	1.13
new products	0.9	0.4	0.8	0.1	1.4	0.67	new products	1.3	0.9	0.3	0.83
association	1.1	-0.5	0.3	0.8	1.4	0.50	pricing	0.7	0.5	0.3	0.50
pricing	0.5	0.4	0.0	0.3	1.0	0.42	association	0.2	1.1	-0.2	0.37
organizational structuring	-0.1	-0.4	0.7	-0.1	-0.2	0.00	adoption of standards and rules	0.0	0.0	-0.1	-0.03
training	-0.2	0.0	0.4	0.4	-1.1	-0.07	training	0.1	-0.2	-0.0	-0.03
volunteerism	0.0	-0.1	0.2	0.2	-0.7	-0.10	organizational structuring	-0.3	-0.1	-0.1	-0.17
adoption of standards and rules	0.0	-0.3	-0.2	0.0	0.1	-0.10	volunteerism	-0.5	0.0	-0.3	-0.27
modification of procedures	-1.9	0.5	3.0	-4.0	-2.9	-0.85	assessment and measurement	-2.0	-1.4	-1.1	-1.50
assessment and measurement	-1.4	-0.5	-1.4	-1.3	-1.4	-1.15	modification of procedures	-3.2	-1.9	-0.6	-1.90
asset modification	-4.1	-1.0	-3.6	-1.9	-5.7	-3.05	asset modification	-3.8	-4.1	-3.8	-3.90

	Full	Q_1	Q_2	Q_3	Q_4	Average		2018	2019	2020	Average
SDG 7	10.2	2.5	1.6	10.8	16.3	8.28	SDG 7	12.1	10.2	5.3	9.20
SDG 15	0.2	3.0	-0.1	-1.1	0.8	0.56	SDG 11	0.1	-0.2	0.1	0.00
SDG 13	0.1	0.6	-0.6	-0.1	0.4	0.08	SDG 13	0.0	0.1	-0.2	-0.03
SDG 14	-0.2	-0.3	0.0	-0.1	-0.3	-0.18	SDG 14	-0.2	-0.2	0.1	-0.10
SDG 11	-0.2	-1.2	-0.2	0.3	-0.2	-0.30	SDG 15	-0.9	0.2	0.2	-0.17
SDG 6	-1.2	-1.9	1.3	-0.6	-2.5	-0.98	SDG 6	-1.2	-1.2	-0.2	-0.87
SDG 12	-9.0	-1.7	-1.8	-8.5	-14.8	-7.16	SDG 12	-9.5	-9.0	-6.0	-8.17

Table ST12: **Excess effort across size and estimation windows (well below 2°).** The table show the excess effort across size and estimation windows for a well below 2°C target. Positive values denote actions and SDGs more prevalent in the aligned population.

Topic	0
waste management	use,reduce,product,emission,carbon,waste,energy,supplier,water,material,site,environmental,program,sustainability,impact
energy efficiency	energy,consumption,system,light,lighting,reduce,lead,saving,use,building,air,efficiency,install,efficient,save
stakeholder orientation	support,employee,community,project,school,climate,initiative,local,provide,change,year,program,green,people,environmental
renewable energy	energy,power,solar,customer,electricity,renewable,program,project,provide,efficiency,home,use,help
fleet & travel	emission,reduce,vehicle,fuel,use,carbon,car,transport,fleet,electric,travel,employee,year,engine,truck
plant management	plant,emission,water,reduce,gas,use,energy,system,production,process,waste,project,air,heat,facility
environmental planning	activity,area,consumption,development,employee,energy,environmental,green,group,management,office,power,project,promote,reduce,sustainable,system,use,work

Table ST13: **Topic analysis** The table shows the words associated with each of the topic identified in the sustainability reports.

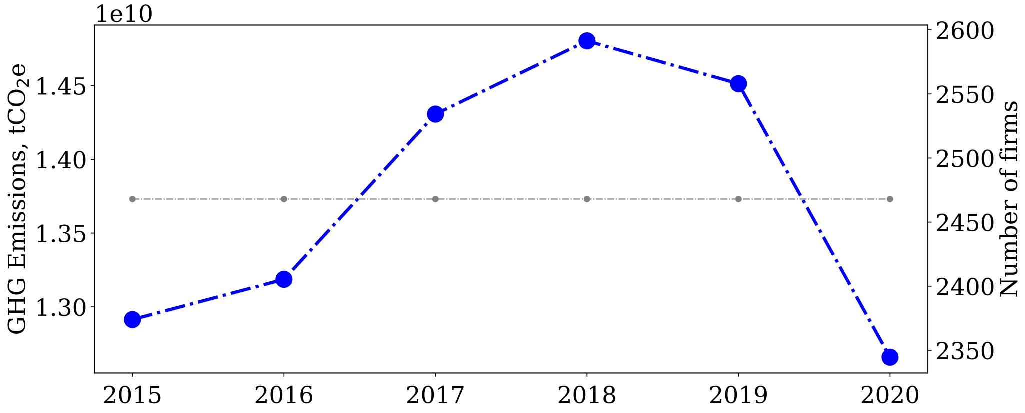

Fig. S15 | Emissions have continued to rise since 2015. The figure shows the average emissions of companies (blue line) for which emissions were available every year since 2015 (gray line). The figure shows that total GHG emissions have been steadily rising, except for 2020. The drop of 2020 emissions is likely due to lockdown measure introduced to curb the spreading of COVID-19. Indeed, in our sample we observe a drop of $\sim 9\%$ to be compared with an estimated drop of $\sim 6.4\%$ globally as reported in Tollefson, J. *COVID curbed carbon emissions in 2020 — but not by much*, Nature 589, 343 (2021). The figure illustrates the importance of removing 2020 from our sample in order to reduce the impact of confounding effects in our analysis.

Below 2°C Size$_q$	Magnitude of deviation Negative excess effort	Magnitude of deviation Positive excess effort	Probability of alignment Negative excess effort	Probability of alignment Positive excess effort
0	0.0	-0.02***	0.0	0.1*
1	-0.0	-0.02**	0.04	0.06
2	0.01	-0.02***	0.0	0.08
3	0.0	-0.01	0.01	0.09*
4	0.0	-0.02***	0.0	0.09*

Below 2°C Year	Magnitude of deviation Negative excess effort	Magnitude of deviation Positive excess effort	Probability of alignment Negative excess effort	Probability of alignment Positive excess effort
2018	-0.01	-0.02**	0.08	0.1
2019	0.0	-0.02***	0.0	0.1*
2020	-0.01	-0.02***	-0.02	0.11**

Well Below 2°C Size$_q$	Magnitude of deviation Negative excess effort	Magnitude of deviation Positive excess effort	Probability of alignment Negative excess effort	Probability of alignment Positive excess effort
0	0.01	-0.02***	-0.03	0.14***
1	0.01*	-0.01**	-0.03	0.09*
2	0.01**	-0.0	-0.04	0.09*
3	0.0	-0.02***	-0.03	0.12**
4	0.01	-0.02***	-0.02	0.14***

Well Below 2°C Year	Magnitude of deviation Negative excess effort	Magnitude of deviation Positive excess effort	Probability of alignment Negative excess effort	Probability of alignment Positive excess effort
2018	-0.0	-0.01	-0.05	0.12
2019	0.01	-0.02***	-0.03	0.14***
2020	-0.0	-0.01***	-0.08	0.06

Table ST14: **Robustness of the effect across size quartiles and estimation windows without non- causative actions.** The tables show the estimation of the association between initiatives in positive (blu) and negative(red) excess effort with the magnitude and probability of alignment with climate targets after excluding non-causative activity types (volunteerism and donation&funding). The tables show that results are mostly unchanged supporting the hypothesis of the existence of a link between initiatives and emission reduction. However, non-causative initiatives add some explanatory value. The effects in the different estimation windows are estimated over the full populations. *,**,*** denote statistical significance at the 10%,5%, and 1% level, respectively, calculated from the two-tailed p-values for the t-statistics of the parameters.

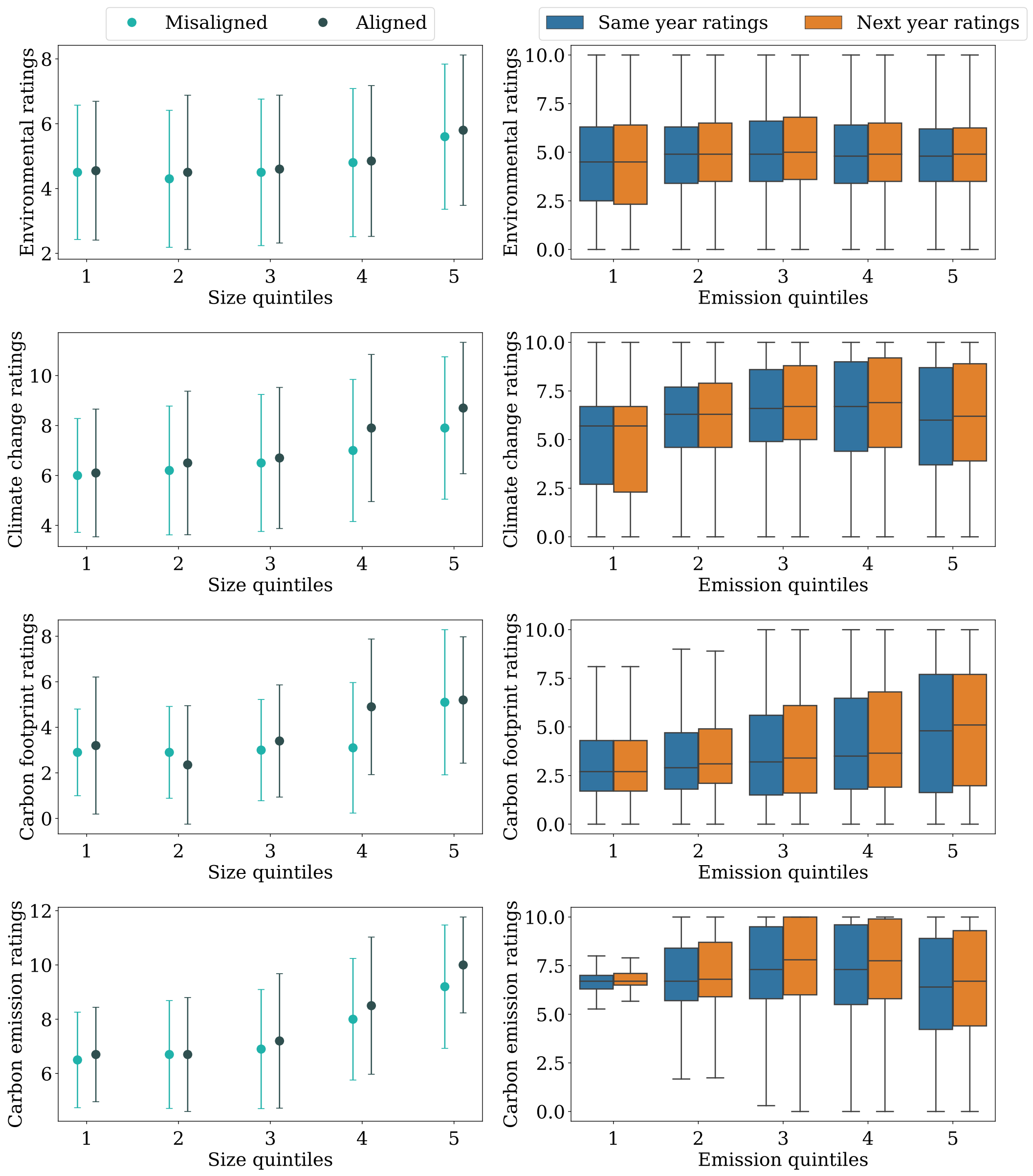

Fig. S16 | Flaw of ESG ratings. The left columns show the average value of the environmental ratings and its sub-scores (from MSCI) of companies with emission pathways aligned and misaligned with the target set by the Paris agreement (error bars show standard deviations). The right columns show the distributions of the same ratings and sub-scores as function of the absolute value of the emissions (in quintiles). The line of the box plots are median lines, the edges of the boxes are the quartile range: the 25th and 75th percentile. Overall the figure shows that environmental ratings and sub-scores fail in capturing the alignment with climate targets (**a**) and the absolute value of emissions (**b**).

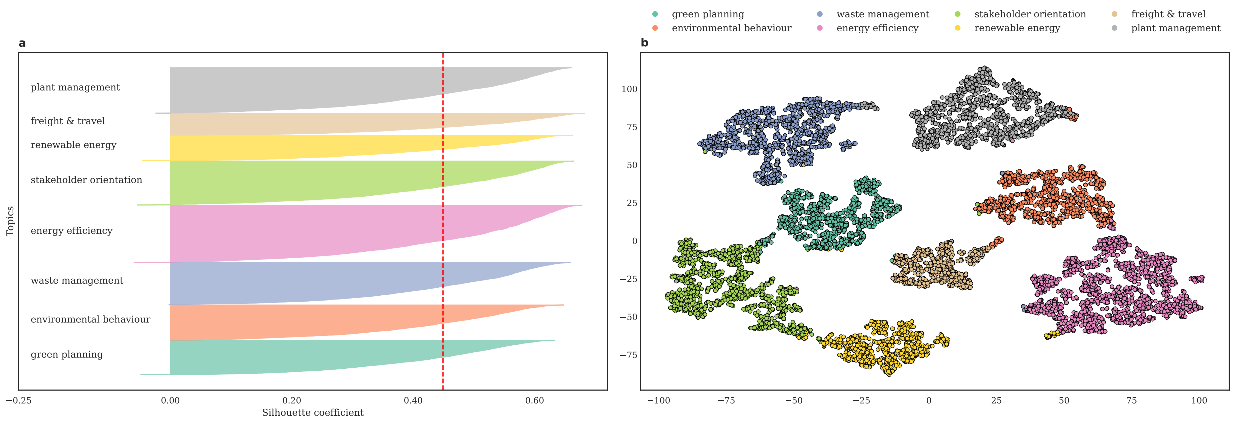

Fig. S17 | Topics overlap and distinctiveness. The Figure shows the results of our robustness test on the finding of the topic analysis, Figure 6 in the main text. Panel **a** shows the silhouette scores of initiatives as clustered by their dominant topic (i.e., assigned to a topic if it is their strongest association and with more than 35% probability overall). Silhouette scores compare the relative distance of initiatives from their own and neighbouring topics. They range from values of -1 to 1, with negative values pointing to likely misclassified initiatives. The vertical line shows the in-sample average of the silhouette score. Panel **b** shows initiatives clustered by dominant topic and projected onto a $2D$ space through a T-distributed stochastic neighbor embedding method. The relative between and within distances of the topics indicate their distinctiveness and coherence, respectively. Overall, the figure shows that the identified topics are well separated (have minimal overlap).

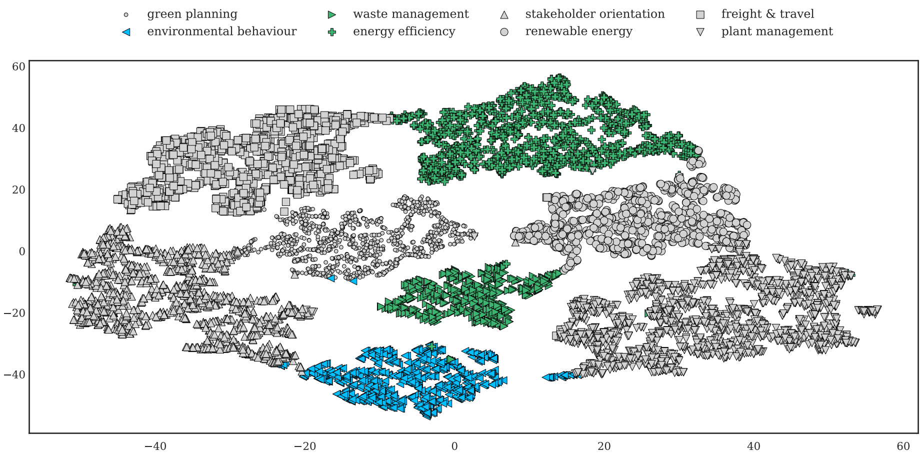

Fig. S18 | Distinctiveness of aligned and misaligned topics. We use the t-SNE analysis (see figure S17) to represent the distinctiveness between topics relatively more prevalent in initiatives of misaligned companies or aligned ones. The figure shows initiatives according to their dominant topic through the use of markers, while color codes are used to identify the topics statistically overrepresented in initiatives from misaligned companies (green), aligned companies (blue), as well as statistically indistinguishable topics (grey) in accordance to the analysis shown in figure 6. Overall, the figure shows that topic overrepresented in the two groups (aligned and misaligned companies) are substantially different.
