# Supplementary material for: The alignment of companies' sustainability behavior and emissions with global climate targets
Source: Nat Commun. 2023 Dec 5;14:7831. doi: 10.1038/s41467-023-43116-2 (PMC10698019; doi:10.1038/s41467-023-43116-2)
Supplement: Supplementary file 3 — Reporting Summary [file 41467_2023_43116_MOESM3_ESM.pdf]

nature portfolio

Corresponding author(s): Simone Cenci, Maurizio Zollo

Last updated by author(s): 19/10/2023

Reporting Summary

Nature Portfolio wishes to improve the reproducibility of the work that we publish. This form provides structure for consistency and transparency in reporting. For further information on Nature Portfolio policies, see our Editorial Policies and the Editorial Policy Checklist.

Statistics

For all statistical analyses, confirm that the following items are present in the figure legend, table legend, main text, or Methods section.

n/a	Confirmed	
☐	☒	The exact sample size (n) for each experimental group/condition, given as a discrete number and unit of measurement
☐	☒	A statement on whether measurements were taken from distinct samples or whether the same sample was measured repeatedly
☐	☒	The statistical test(s) used AND whether they are one- or two-sided<br>*Only common tests should be described solely by name; describe more complex techniques in the Methods section.*
☐	☒	A description of all covariates tested
☐	☒	A description of any assumptions or corrections, such as tests of normality and adjustment for multiple comparisons
☐	☒	A full description of the statistical parameters including central tendency (e.g. means) or other basic estimates (e.g. regression coefficient) AND variation (e.g. standard deviation) or associated estimates of uncertainty (e.g. confidence intervals)
☐	☒	For null hypothesis testing, the test statistic (e.g. F, t, r) with confidence intervals, effect sizes, degrees of freedom and P value noted<br>*Give P values as exact values whenever suitable.*
☒	☐	For Bayesian analysis, information on the choice of priors and Markov chain Monte Carlo settings
☒	☐	For hierarchical and complex designs, identification of the appropriate level for tests and full reporting of outcomes
☒	☐	Estimates of effect sizes (e.g. Cohen's d, Pearson's r), indicating how they were calculated

Our web collection on statistics for biologists contains articles on many of the points above.

Software and code

Policy information about availability of computer code

Data collection	Data was collected from publicly available sustainability reports. PDF are converted into plain text using pdftotext in Python3.8
Data analysis	Python3.8. The code is available on Harvard Dataverse at https://doi.org/10.7910/DVN/C7ILED

For manuscripts utilizing custom algorithms or software that are central to the research but not yet described in published literature, software must be made available to editors and reviewers. We strongly encourage code deposition in a community repository (e.g. GitHub). See the Nature Portfolio guidelines for submitting code & software for further information.

Data

Policy information about availability of data

All manuscripts must include a data availability statement. This statement should provide the following information, where applicable:

- Accession codes, unique identifiers, or web links for publicly available datasets
- A description of any restrictions on data availability
- For clinical datasets or third party data, please ensure that the statement adheres to our policy

The sustainability behaviour dataset is available on Harvard Dataverse at https://doi.org/10.7910/DVN/C7ILED
Emissions and accounting data are covered by a license and therefore they cannot be shared by us. However, they can be accessed directly from the data providers for a fee, see Refs. 40-44 for the links to the data providers' websites

Research involving human participants, their data, or biological material

Policy information about studies with human participants or human data. See also policy information about sex, gender (identity/presentation), and sexual orientation and race, ethnicity and racism.

Reporting on sex and gender	N/A
Reporting on race, ethnicity, or other socially relevant groupings	N/A
Population characteristics	N/A
Recruitment	N/A
Ethics oversight	N/A

Note that full information on the approval of the study protocol must also be provided in the manuscript.

Field-specific reporting

Please select the one below that is the best fit for your research. If you are not sure, read the appropriate sections before making your selection.

☐ Life sciences ☒ Behavioural & social sciences ☐ Ecological, evolutionary & environmental sciences

For a reference copy of the document with all sections, see nature.com/documents/nr-reporting-summary-flat.pdf

Behavioural & social sciences study design

All studies must disclose on these points even when the disclosure is negative.

Study description	In this study we perform an empirical analysis of quantitative data on corporate disclosure of sustainability initiatives to estimate the relation between corporate behaviour and alignment with climate targets
Research sample	The full sample include 4191 publicly traded companies. The sampled analysed in the main analysis include 1951 publicly traded companies in the hard-to-abate and energy-intensive sectors, and 26944 climate related sustainability initiatives between 2010-2020.
Sampling strategy	Our sample includes all the publicly traded companies for which we could collect a sustainability or integrated report.
Data collection	The data were collected in three batches. First we retrieve links to published sustainability reports from Refinitiv Asset4, a major provider of ESG financial data. Second, we purchased the remaining documents from Corporate Register, the largest provider of historical sustainability reports. Finally, we crawled the remaining documents from Google using Python3.8.
Timing	Data were collected from May 2022 to February 2023
Data exclusions	No data were excluded in the descriptive analysis. In the regression analysis we excluded companies for which we only had less than two years of observations
Non-participation	N/A
Randomization	This is an empirical study and does not require randomisation

Reporting for specific materials, systems and methods

We require information from authors about some types of materials, experimental systems and methods used in many studies. Here, indicate whether each material, system or method listed is relevant to your study. If you are not sure if a list item applies to your research, read the appropriate section before selecting a response.

Materials & experimental systems

n/a	Involved in the study
☒	☐ Antibodies
☒	☐ Eukaryotic cell lines
☒	☐ Palaeontology and archaeology
☒	☐ Animals and other organisms
☒	☐ Clinical data
☒	☐ Dual use research of concern
☒	☐ Plants

Methods

n/a	Involved in the study
☒	☐ ChIP-seq
☒	☐ Flow cytometry
☒	☐ MRI-based neuroimaging
